# Syndecan-4 is a Novel Therapeutic Target for Intervertebral Disc Degeneration via Suppressing JNK/p53 Pathway

**DOI:** 10.7150/ijbs.40189

**Published:** 2020-01-14

**Authors:** Jun Ge, Xiaoqiang Cheng, Chenxi Yuan, Jiale Qian, Chunshen Wu, Cheng Cao, Huilin Yang, Feng Zhou, Jun Zou

**Affiliations:** Department of Orthopaedic Surgery, The First Affiliated Hospital of Soochow University, Suzhou, Jiangsu 215006, China

**Keywords:** Syndecan-4, disc degeneration, JNK, p53, signal pathway

## Abstract

Syndecan-4 is a member of the polysaccharide syndecan family and plays a vital role in intervertebral disc development. Several studies have demonstrated the positive relationship between syndecan-4 expression and intervertebral disc degeneration. However, the detailed molecular mechanism by which syndecan-4 affects the degeneration of nucleus pulposus cells (NPCs) remains unclear. In this study, cell viability was determined by CCK-8 assay, mRNA level was determined by qPCR, and protein expression was determined by western blot. Molecular interaction was determined by chromatin immunoprecipitation assay. A rabbit intervertebral disc degeneration model was established to test for syndecan *in vivo*. We found that the morphology and viability of NPCs were not affected by the expression of syndecan-4 in the long term. While the NPC function were affected, which results in the degeneration of intervertebral disc. Syndecan-4 overexpression promoted the degeneration of NPCs. Syndecan-4 also activated the JNK signaling pathway and downstream p53 pathways, and promoted degeneration. Inhibition of the JNK pathway, which down-regulated p53 expression, alleviated the degeneration. In an *in vivo* study, syndecan-4 siRNA injection stopped the development of rabbit disc degeneration, and even created a reverse effect, in which JNK/p53 played a role. Syndecan-4 may be a novel therapeutic target for intervertebral disc degeneration via suppressing the JNK/p53 pathway.

## Introduction

Intervertebral disc degeneration (IVD) can lead to protrusion of intervertebral discs, spinal instability, spinal stenosis, and other degenerative diseases that can cause severe back pain and reduced mobility 1. Current surgical and non-surgical treatments do not target the pathological changes induced by disc degeneration, but are mainly supportive treatments aimed at relieving symptoms 2, 3. As society includes a growing number of older people, the incidence of disc degenerative diseases is gradually rising. Accordingly, novel therapeutics that can effectively prevent and treat disc degenerative diseases are urgently needed 4, 5. In recent years, with increased understanding of intervertebral disc biology and rapid development of genetic engineering technology, researchers have explored applications of localized gene therapy in the intervertebral discs, which would increase the efficacy treatment while reducing side effects on other tissues and organs 6, 7.

Syndecan-4 is a member of the polysaccharide syndecan family, which belongs to the family of transmembrane heparan sulfate proteins 8, 9. Vascular endothelial cells and smooth muscle cells are two main sites of syndecan-4 expression. It is a growth factor-binding co-receptor that regulates a variety of cell biological functions and plays important roles in cell spreading, recognition, adhesion, migration, and proliferation. It also mediates inflammatory reaction 10, 11. Recent studies have shown that syndecan-4 protein is altered in inflammation, wound healing, and a variety of diseases. In combination with the intercellular matrix and growth factors, syndecan-4 regulates downstream signaling pathways, thereby affecting multiple physiological functions 12, 13. Echtermeyer *et al.* demonstrated that syndecan-4 is a vital factor in cartilage growth and development 14. During the process of articular cartilage degeneration, syndecan-4 inhibits the differentiation and repair of articular cartilage as well as the synthesis of cartilage extracellular matrix.

Beckett et al. suggested that syndecan-4 plays an important role in the formation of the annulus fibrosus of intervertebral discs, and especially in the production of actin cytoskeleton and nascent extracellular matrix 15. The study confirms the proliferation and migration functions of syndecan-4 in intervertebral discs. Several recent studies have revealed a positive relationship between high syndecan-4 expression and IVD. Wang et al. found that TNF-α and IL-1β promote expression of syndecan-4 in nucleus pulposus cells (NPCs) through the NF-κB pathway, enhance the activity of a disintegrin and metalloprotease with thrombospondin type I motifs (ADAMTS-5), and cause the degradation of the major protein components of intervertebral discs^16^. Further study suggested that expression of syndecan-4 in NPCs is controlled by hypoxia-inducible factor 1α and prolyl-4-hydroxylase domain protein 2. In addition, Sox9, an important factor for the synthesis of aggrecan and collagen II, was also found to be controlled by the heparan-sulphate side chains of syndecan-4^17^. Research by Yang et al. demonstrates the protective role of TGF-β1 in IVD by inhibiting the expression of syndecan-4^18^. Generally speaking, studies on the upstream signaling pathway of syndecan-4 have been robust. However, these studies were all *in vitro*, and no *in vivo* experiments were conducted. From our point of view, as syndecan-4 is an important developmental factor for intervertebral discs, its inhibition by inhibiting upstream signaling pathways will disrupt intervertebral discs in some way. It is therefore urgent to examine the detailed underlying molecular mechanism of syndecan-4 on the degeneration of NPCs in order to create a breakthrough in the molecular biotherapy of IVD.

Several studies on non-intervertebral disc tissue have shown that syndecan-4 exerts its function through its influence on many pathways. Syndecan-4 affects the ERK signaling pathway through interacting with PKC-α 19. Syndecan-4 also affects Rho and RAF signaling pathways by interacting with β-integrin 20. In addition, Notch signaling pathway and syndecan protein family members have synergistic functions in muscle cell generation 21, suggesting multiple functions for syndecan-4 protein. Syndecan-4 also controls the chondrocyte phenotype via WNT signaling pathways 22. Thus, syndecan-4 may be a significant regulator in many signaling pathways.

The molecular mechanism of syndecan-4 protein in NPC degeneration remains unclear, and no relevant study, including *in vivo* experiments, has been reported. Understanding the underlying mechanism is of great significance for developing accurate therapeutics to treat IVD. This study aimed at revealing the mechanism underlying syndecan-4-mediated IVD, thus providing a potential new therapeutic target for IVD. We investigated the role of syndecan-4 in NPC degeneration in intervertebral discs at the cellular and molecular levels utilizing NPC culture, gene overexpression and knockdown, and an IVD animal model.

## Material and methods

### Culture of human nucleus pulposus cell line

Human nucleus pulposus cells (HNPC) were obtained from ScienCell Research Laboratories (Catalog No. 4800). A vial of HNPC was moved from liquid nitrogen and rapidly thawed in a 37 ºC water bath. Cells were then gently resuspended in 5 mL of medium. The vial was centrifuged at 1,500 rpm for 5 min. After supernatant was removed, cells were resuspended in 10 mL of medium and seeded in a T-75 flask. The culture medium was changed after the first 24 h and then every 2 days afterward. Cells were passaged at 1:2 upon reaching 90% confluence.

### Construction of syndecan-4 overexpressing and knocked-down nucleus pulposus cells

The full-length human syndecan-4 gene was amplified by PCR and subcloned into pMSCV-PIG plasmid for expression as a green fluorescent protein (GFP) fusion protein. Syndecan-4 inhibiting gene was subcloned into FG12 plasmid with GFP. The constructed plasmids were kind gifts from the Chinese Academy of Science. Insertion of the gene was verified by restriction endonuclease analysis and sequencing. The pMSCV-PIG-syndecan-4 and FG12-knockdown-syndecan-4 plasmids were then transfected into NPCs. GFP-positive cells were sorted and collected by flow cytometry.

### Evaluation of nucleus pulposus cell function

Syndecan-4 overexpressing cells, knocked-down cells, and control cells (normal cells) were cultured for 4 days. Changes in cell morphology were recorded using an inverted microscope during this period. Cell viability was tested using the Cell Counting Kit-8 (CCK-8, Dojindu, Japan) on the 1^st^, 2^nd^, 3^rd^, and 4^th^ day. At the end of the 4^th^ day, total RNA and protein were extracted from each group, and mRNA and protein levels of aggrecan, collagen II, collagen X, and Sox-9 were measured by quantitative PCR and western blotting, respectively.

### qPCR

After cells were washed with PBS three times, 1 mL TRIzol (Gibco, CA, USA) was used for cell lysis. After that, RNA was further extracted with a TRIzol RNA extraction kit (Ambion, TX, USA). RNA samples were then quantified to equivalent and finally reverse-transcribed into first-strand cDNA (Thermo Scientific, CA, USA). The 20 μL reaction mixture consisted of 10 μL of SYBR green mixture, 1 μL of each primer (10 μmol/L, primers' sequences are in Table 1), 1 μL of cDNA template, and 7 μL of ddH_2_O. The mixture was then incubated at 95 °C for 10 min, then cycled 40 times at 95 °C for 15 s, 60 °C for 30 s, and 72 °C for 30 s. For each sample, the relative amount of target mRNA was determined and normalized to β-actin. Data are presented as the fold change (2^-ΔΔCT^). Finally, all data were compared with those of the the control group to obtain relative mRNA expression levels.

### Western Blot

After three washes with PBS, cells were suspended in 4 °C cell lysis buffer (Bio-Rad, CA, USA). Proteins in the lysates were quantified to equivalent and separated by 12% SDS-PAGE. They were then transferred to nitrocellulose membranes (Bio-Rad, CA, USA). Membranes were blocked with 5% non-fat dry milk (Yili, China) in Tris-buffered saline Tween-20 buffer (Thermo Fisher Scientific, MA, USA). After blocking, membranes were incubated with primary antibodies at 4 °C overnight. For western blotting primary antibodies, anti-Sox-9, anti-p53, and anti-JNK were purchased from Cell Signaling Technology (MA, USA). Anti-aggrecan was purchased from Santa Cruz Biotechnology (TX, USA). Anti-collagen-X and anti-collagen-II were purchased from Abcam (CA, USA). After primary antibody incubation, membranes were fully washed and incubated with secondary antibodies (Beyotime, China) at room temperature for 1 h. Finally, membranes were visualized with ECL Prime (Thermo Scientific, CA, USA). GAPDH expression level was used as an internal control.

### Construction of p53 overexpressing nucleus pulposus cells

Full-length p53 gene was cloned into pMSCV-PIG plasmid with GFP and successfully verified by PCR. The constructed plasmids were kind gifts from the Chinese Academy of Science. The pMSCV-PIG-p53 plasmid was transfected into nucleus pulposum cells via Lipofectamine™ 2000 (Thermo Fisher Scientific, MA, USA), and GFP-positive cells were selected by flow cytometry.

### Signaling pathway analysis

Syndecan-4 overexpressing NPCs, knocked-down cells, and control cells were cultured for 24-72 h. mRNA and protein levels of JNK and p53 (including total protein and phosphorylated protein) were measured by qPCR and western blotting, respectively. Syndecan-4 overexpressing cells were treated with pMSCV-PIG-p53 plasmid and the JNK inhibitor, SP600125. mRNA and protein levels of aggrecan, collagen II, collagen X, and Sox-9 in each group were measured by qPCR and western blotting, respectively.

### Chromatin immunoprecipitation (ChIP) assay

Interactions of p53 with the nucleus pulposus functional proteins, aggrecan, collagen II, and Sox-9 were analyzed by ChIP assay (Millipore, MA, USA). NPCs were cultured in 10-cm dishes. ChIP assay was performed according to the manufacturer's instructions. First, cells were cross-linked with 37% formaldehyde, and DNA was broken into fragments of 200-1000 base pairs (bp) by ultrasound. Mdm2 and p21 were used as positive controls. ChIP-level p53 antibody was used for the Chip assay. IgG antibody was used as a negative control, and the input group was not treated with any antibodies. DNA was extracted through a series of steps, including precipitation, elution, inverse crosslinking, and purification, and served as a template for the real-time PCR assay. The mixture for PCR included 2 μL of DNA extract, 1 μL of 10 μM primers, 7 μL of ddH_2_O, and 10 μL SYBR Green. The initial denaturation step was at 94 ºC for 20 min. The mixture was then cycled 50 times at 94 ºC for 1 min, at 60 ºC for l min and at 72 ºC for 1 min.

### Establishment of a rabbit intervertebral disc degeneration model

One-year-old New Zealand white rabbits (3.0-3.2 kg) were selected for the study (n=18). Rabbits were anesthetized by intramuscular injection of toluenthiazine and ketamine and placed in a lateral decubitus position. Aseptic technique was used for all surgical procedures. The hair over the surgical field of approximately 20 cm × 15 cm was shaved. Through a left retroperitoneal approach, the third lumbar processus transversus (L3) was exposed and removed from the roots, resulting in the exposure of the L3/4 intervertebral disc. This disc was punctured with a 16-gauge needle to a depth of 5 mm from the anterolateral fiber ring for 5 s. The needle was then pulled out without disturbing the spinal cord, and the surgical wound was sutured. Gentamicin (80,000 U) was used before and after surgery. Magnetic resonance imaging (MRI) was used four weeks after surgery to verify the establishment of the IVD model. The discs were scored according to the modified Thompson classification^23^ from grade I to IV (I, normal; II, little decrease of signal intensity but obvious narrowing of high signal area; III, moderate decrease of signal intensity; and IV, severe decrease of signal intensity) in T2-weighted phase. All experimental procedures were performed at the Laboratory Animal Center of Soochow University. All the experimental procedures were approved by the Ethics Committee of the First Affiliated Hospital of Soochow University. All procedures were carried out in strict accordance with the Declaration of Helsinki (1964) and the Laboratory Animal Guidelines for Ethical Review of Animal Welfare (GB/T35892-2018, China).

### Effect of syndecan-4 siRNA injection

After verifying establishment of the disc degeneration model, eighteen New Zealand rabbit models were randomly divided into 3 groups. The disc was exposed again for injection. Twenty microliters of syndecan-4 siRNA (GenePharma, China), control siRNA (GenePharma, China), or saline was injected a group of 6 rabbits using a micro syringe. After injection, the wound was carefully sutured. This was the only injection given during the entire experiment. The MRI performed before injection was recorded as week 0. MRI was performed at 4 and 8 weeks after the injection. Weighted image scanning was used for sagittal T2 lumbar vertebrae. Rabbits were euthanized after the week 8 MRI scan, and the nucleus pulposus of the intervertebral disc tissue was used for H&E staining. The protein expression of collagen II (Abcam, CA, USA), JNK (Santa Cruz, TX, USA), and p53 (Cell Signaling, MA, USA) in the nucleus pulposus tissue was detected by immunohistochemistry. Five regions were randomly selected in each immunohistochemical section, and positive cells were counted with Image-ProPlus 6.0 image analysis software (Media Cybernetics, MD, USA). NPCs in which the nucleus was stained, and the cytoplasm showed brown staining, were considered positive. The average of positive cells was then calculated.

### Statistical analysis

Quantitative data presented as mean ± S.D. were analyzed with one-way ANOVA. The Kruskal-Wallis test was used for non-parametric data. Values of p < 0.05 were considered statistically significant.

## Results

### Neither syndecan-4 overexpression nor knockdown had significant effects on the cell morphology and proliferation of nucleus pulposus cells in the long term

**Figure 1:**
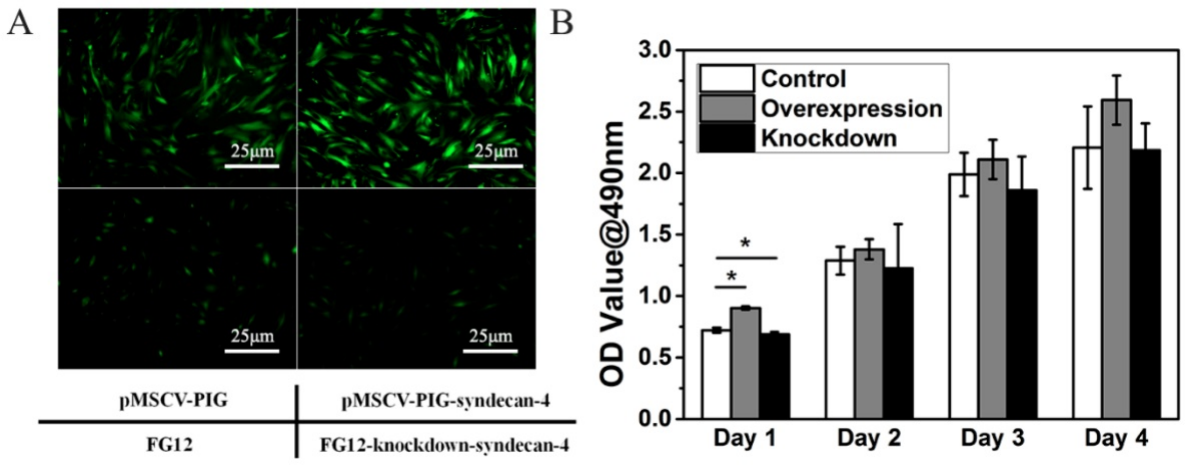
Neither syndecan-4 overexpression nor knockdown had significant effects on the morphology and proliferation of NPCs. A. Syndecan-4 overexpressing or knocked-down NPCs were round, long fusiform, and polygonal, similar to normal NPCs. Morphology was not affected by either overexpression or knockdown of syndecan-4. B. CCK-8 results also showed that cell viability was not affected in the long term.

Within 48 h, the nucleus pulposus cells gradually adhered to the bottom of the culture dish. The cells stretch out pseudopodia, and their morphology changed from oval to polygonal. Gradually and finally, they assembled into the shape of a vortex. Syndecan-4 overexpressing or knocked-down NPCs were similar to normal NPCs. Their morphology was not affected by syndecan-4 overexpression or knockdown, as shown in Figure 1A. However, cells in the overexpression group are slightly more affected than empty loading controls, while the knockdown group shows the opposite result. GFP-positive cells were infected with virus, indicating the high efficacy of infection. CCK-8 results showed that cell viability was slightly higher in syndecan-4 overexpressing cells at days 1, 2, 3, and 4 than that in control cells. Though this observed difference was significant on the 1^st^ day, the difference was not statistically significant in the long term (p > 0.05). Additionally, cell viability was slightly lower in syndecan-4 knocked-down NPCs at days 2, 3, and 4, though with no statistical significance (p > 0.05) (Figure 1B).

### Syndecan-4 negatively regulates nucleus pulposus cell functions

**Figure 2:**
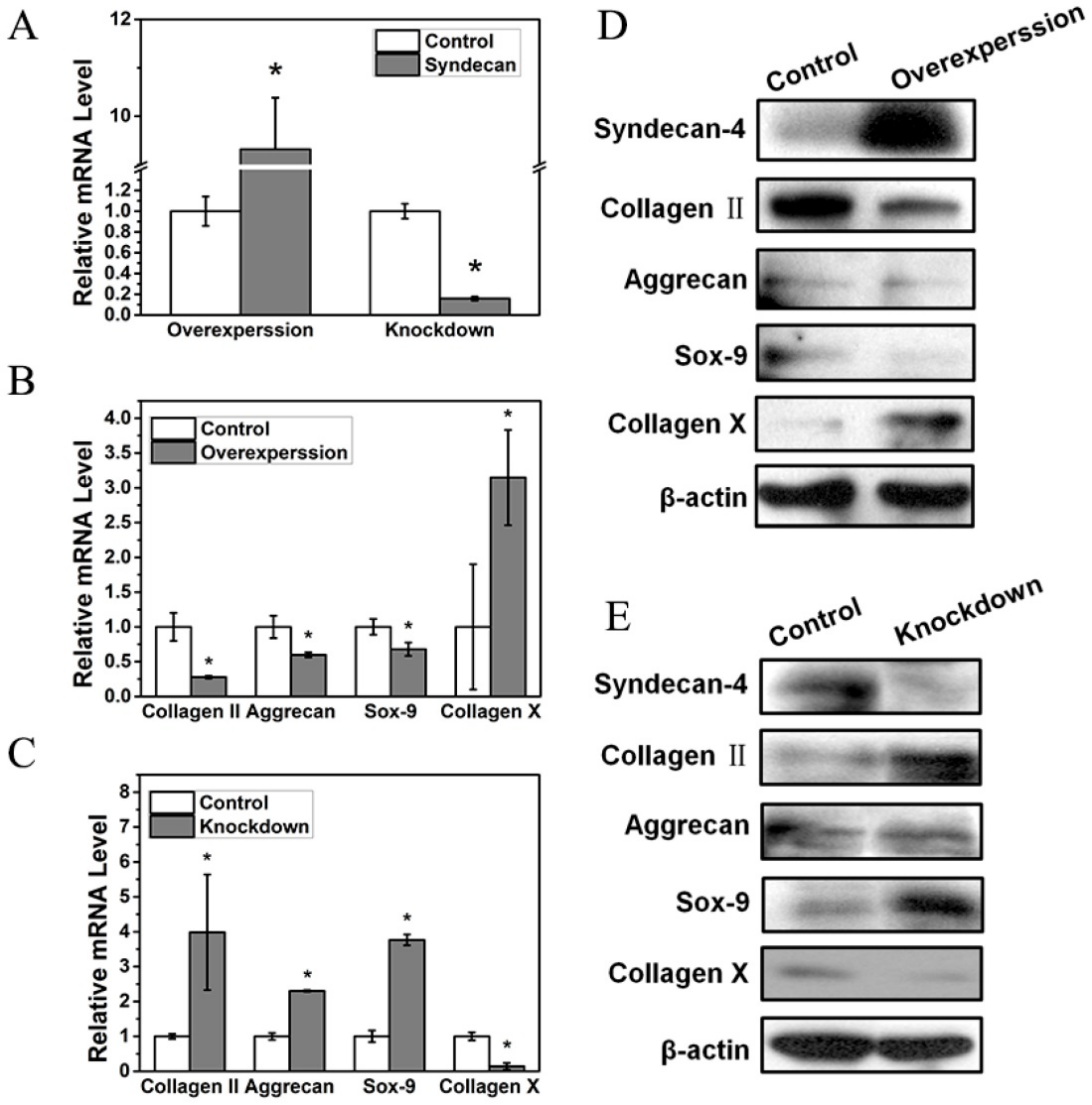
A. Syndecan-4 shows different expression levels in overexpression and knockdown groups. B. mRNA levels of aggrecan, collagen II, and Sox-9 were reduced when syndecan-4 was overexpressed (p < 0.05). Collagen X expression increased significantly (p < 0.05). C. In syndecan-4 knocked-down cells, the expression of aggrecan, collagen II, and Sox-9 significantly increased, and collagen X level was significantly decreased (p < 0.05). D. Western blot showed that aggrecan, collagen II, and Sox-9 expression was clearly lower in syndecan-4 overexpressing cells and collagen X expression was increased in syndecan-4 overexpression group. E. Western blot showed the expression of collagen II, aggrecan and Sox-9 was higher in syndecan-4 knocked-down cells than that in the control group and collagen X was lower in syndecan-4 knockdown group.

In order to further study the influence of syndecan-4 on NPCs in IVD, we measured the functional factors of NPCs overexpressing or knocked down for syndecan-4 (Figure 2A). The mRNA levels of nucleus pulposus functional factors, aggrecan, Sox-9, and collagen II were reduced when syndecan-4 was overexpressed. Meanwhile, the mRNA level of collagen X, a degenerative marker, increased significantly (Figure 2B). In syndecan-4 knocked-down cells, the effects were reversed: aggrecan, collagen II, and Sox-9 levels were significantly increased, and collagen X level was significantly decreased (Figure 2C). Similar to RT-PCR results, western blotting showed that protein levels of aggrecan, Sox-9, and collagen II were significantly lower in syndecan-4 overexpressing cells and higher in syndecan-4 knocked-down cells than those in control cells (Figure 2D and 2E). These results indicate that syndecan-4 may contribute to the degeneration of NPCs in intervertebral discs.

### Syndecan-4 activates the JNK/P53 pathway in nucleus pulposus cells

**Figure 3:**
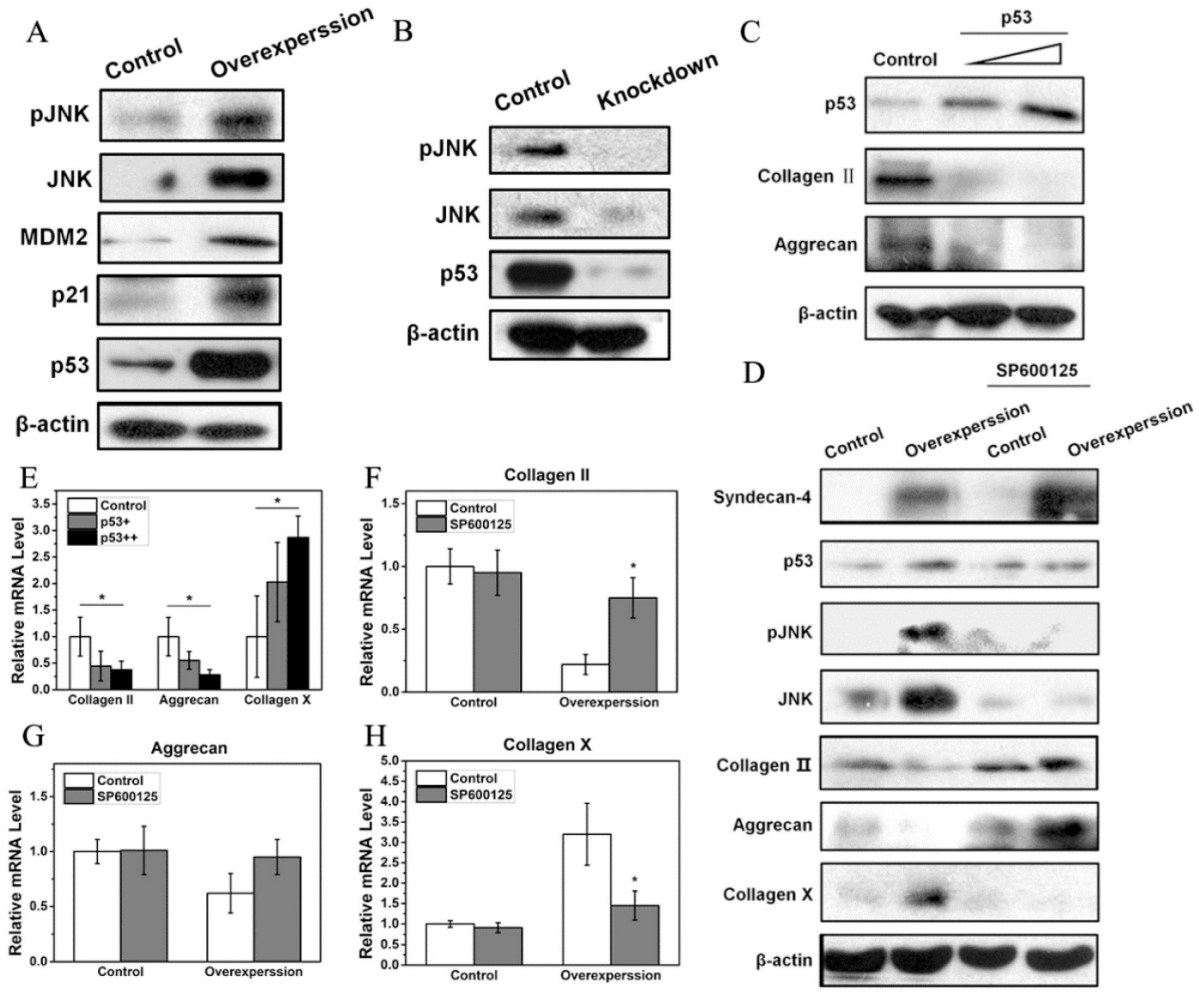
A. Syndecan-4 activates the JNK/p53 pathway in NPCs. Syndecan-4 overexpression significantly increased JNK and pJNK, the expression of p53 and its target genes, MDM2 and p21, was clearly increased. B. Syndecan-4 knockdown decreased the expression levels of pJNK, JNK, and p53. C. After transfection with different concentrations of p53 plasmid, the protein expression of collagen II and aggrecan was decreased which was negatively correlated with concentration of p53 plasmid. D. In cells treated with the JNK inhibitor SP600125, the expression of JNK, pJNK, and p53 was decreased, while the expression of aggrecan and collagen II was increased. In addition, the expression of collagen X get decreased. E. Overexpression of p53 significantly changed the mRNA levels of aggrecan, collagen II, and collagen X. F, G and H. In cells treated with the JNK inhibitor SP600125, the mRNA levels of collagen II, aggrecan and collagen X were significantly changed (p < 0.05).

Western blot results showed that a small amount of JNK and pJNK proteins was observed in normal NPCs. However, syndecan-4 overexpression significantly increased expression levels of JNK and pJNK, while syndecan-4 knockdown significantly decreased the expression levels of both proteins, suggesting that syndecan-4 can activate JNK signaling pathway in NPCs of intervertebral discs. Western blot results also showed that, in syndecan-4 overexpressing NPCs, expression levels of p53, a downstream protein of JNK, and its target genes, MDM2 and p21, were increased (Figure 3A). However, in syndecan-4 knocked-down cells, the expression of p53 was undetectable (Figure 3B). When p53 was overexpressed, both western blot and RT-PCR showed that the protein and mRNA levels of collagen II and aggrecan decreased significantly compared to that of the control cells. On the opposite, mRNA expression of collagen X was significantly increased (Figure 3C and 3E). When cells were treated with the JNK inhibitor SP600125, western blot showed that the expression of pJNK, and p53 proteins are almost the same as that in the control groups. Protein level of JNK is even lower than that in control group. While protein expression of collagen II and aggrecan was increased and collagen X protein expression was decreased when SP600125 was applied (Figure 3D). However, when syndecan-4 was overexpressed, mRNA expression levels of aggrecan, collagen II increased and collagen X was decreased when JNK inhibitor was applied; compared with those in the control cells (Figure 3F, G and H).

### MDM2, p21 and aggrecan are target genes for p53

**Figure 4:**
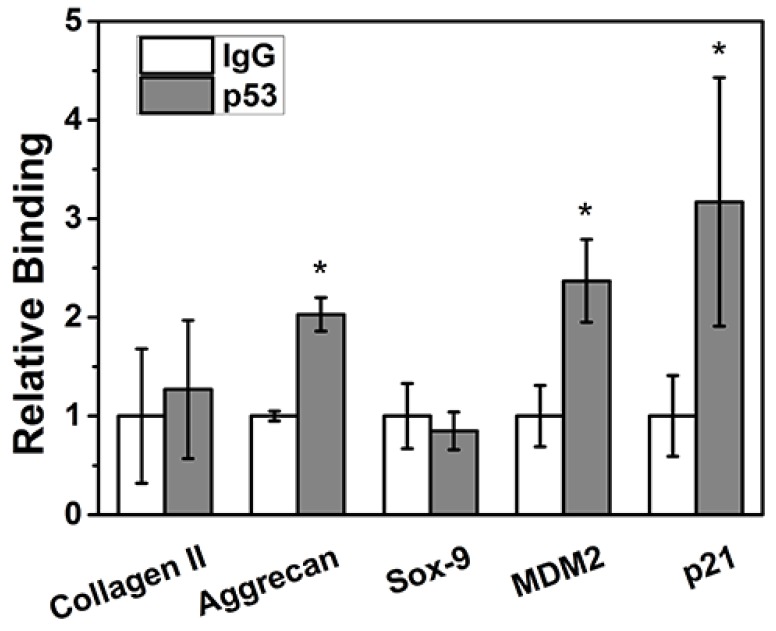
Aggrecan was bound to the p53 site. ChIP assay showed that p53 protein could bind to the promoter regions of the positive control genes MDM2 and p21. After p53 treatment, aggrecan mRNA levels were significantly increased (p < 0.05); however, no obvious change was detected in Sox9 or collagen II expression (p > 0.05).

ChIP assay showed that p53 protein could bind to the promoter regions of the positive control genes MDM2 and p21 (p < 0.05) in NPCs to significantly regulate their transcriptional expression. In terms of the NPC function genes, aggrecan can be directly transcriptionally regulated by p53 (p < 0.05); however, Sox9 and collagen II sites cannot be transcriptionally regulated by p53 (p > 0.05) (Figure 4).

### Syndecan-4 siRNA delays rabbit intervertebral disc degeneration

**Figure 5:**
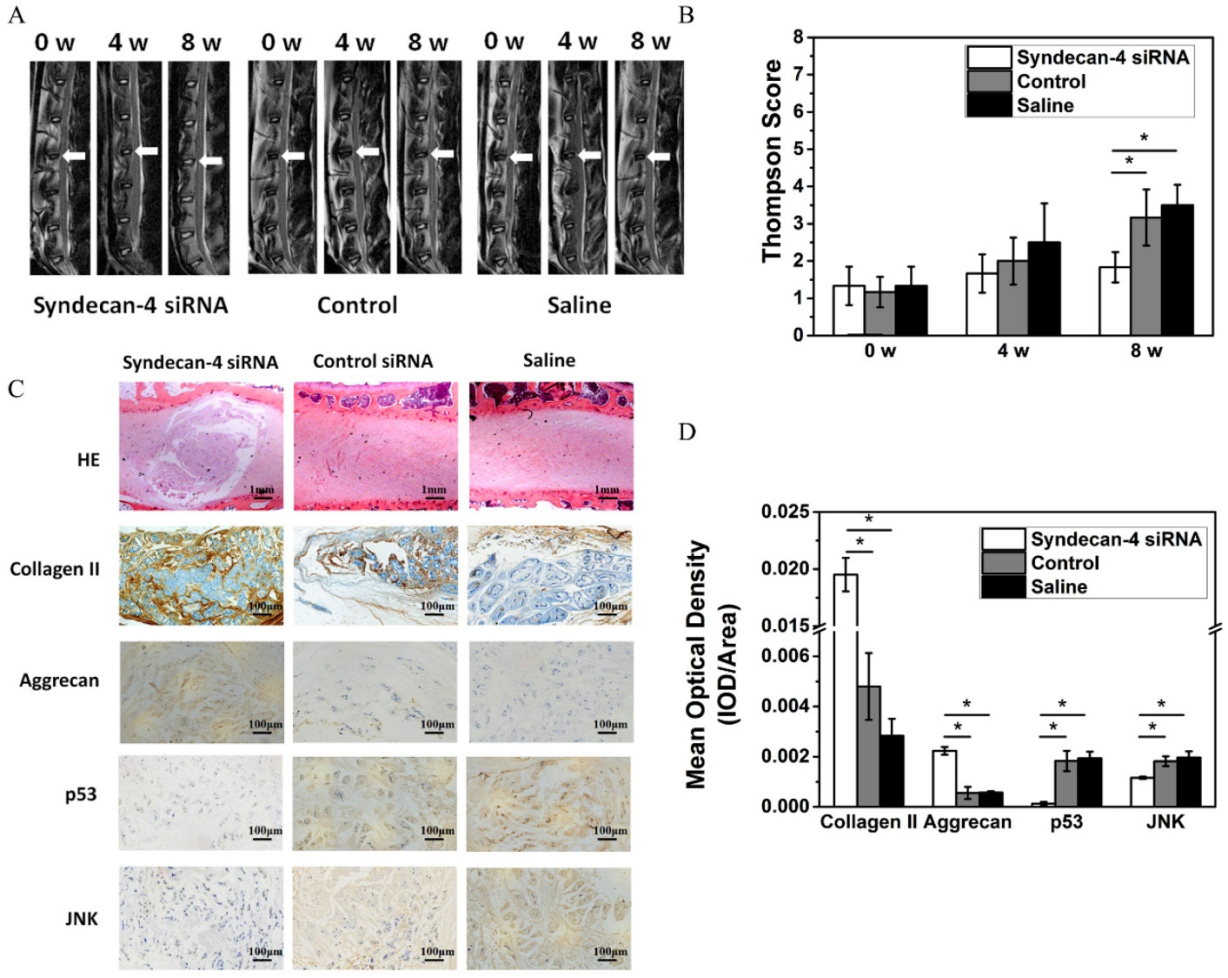
A. Magnetic resonance imaging findings after syndecan-4 siRNA injection in rabbit intervertebral discs. T2 signal intensity was stronger in the syndecan-4 siRNA-injected discs than in control discs. B. Quantification of Thompson score. C. Histological analysis of the intervertebral discs by hematoxylin and eosin staining showed that many NPCs were observed in the syndecan-4 siRNA group. Many NPCs were replaced by fibrocartilaginous tissue in the two control groups. Immunohistochemical staining showed that collagen II and aggrecan were detected in the syndecan-4 siRNA group at 8 weeks. p53 and JNK immunohistochemical staining showed that both p53 and JNK expression levels were lower in the syndecan-4 siRNA group at 8 weeks. D. Quantification of immunohistochemical staining, indicating that syndecan-4 siRNA accelerates the degeneration process via the JNK/p53 pathway.

Eight weeks after injection, MRI results showed that the T2 weighted images on intervertebral discs in the syndecan-4 siRNA group had significantly higher signal intensity than that in control and saline control groups (Figure 5A). The results of the modified Thompson grading evaluation of rabbit lumbar degeneration are presented in Figure 5C. When compared with the control or saline group, the syndecan-4 siRNA group presented significantly lower grades (p < 0.05), while no significant difference was found between the control and the saline groups (p > 0.05). Histological observation showed that the number of visible NPCs decreased in both the control and saline groups (Figure 5B). Scar tissue hyperplasia around the nucleus pulposus and matrix degeneration were observed. However, in the syndecan-4 siRNA group, the annulus fibrosus was arranged in an orderly manner, NPCs were decreased, and the mesh-like structure in the nucleus pulposus, indicating the formation of collagen, was slightly fuzzy. The amounts of proteoglycan and water were reduced. The nucleus pulposus and annulus fibrosus boundary area were clear. Immunohistochemistry results showed that samples from the syndecan-4 siRNA group were positively stained for type II collagen and aggrecan. In the syndecan-4 siRNA group, the extracellular matrix of the nucleus pulposus was stained, and the color was intense, with higher density, while in the control and saline groups, the staining of extracellular matrix became lighter, suggesting that syndecan-4 siRNA delays the IVD process in the model. JNK and p53 immunostaining was mainly observed in the intervertebral disc cell cytoplasm. Immunoreactive cells were mostly located in the nucleus and the inner annulus, and positive staining was observed as brownish-yellow granules in cells. The rate of nucleus pulposus JNK and P53 immuno-positive cells is presented in Figure 5C. Significant differences were detected among the groups (Figure 5D, p < 0.05). The number of JNK and p53 immunoreactive cells in the syndecan-4 siRNA group was low, and a significant difference was detected in the percentage of positive cells when compared with that in the control and saline groups (p < 0.05).

## Discussion

An important transmembrane protein, syndecan-4 is not only a crucial signal transmitter from the cell surface to the inside of the cell, but also an important presenter in response to extracellular signals to induce cell proliferation^13^. To study the effect of syndecan-4 on NPCs of intervertebral discs, we constructed a virus system to overexpress and knock down syndecan-4, and we used it to infect NPCs. First, we investigated the effect of syndecan-4 on the morphology of NPCs. Our results showed that syndecan-4 overexpression or knockdown did not affect the morphology of NPCs in intervertebral discs. Then, we used CCK-8 assay to detect cell proliferation. Syndecan-4 overexpression or knockdown had no significant effect on the proliferation rate of NPCs compared to that in the control group in the long term, indicating that changes in the expression of syndecan-4 do not affect the viability of NPCs over time. Degeneration of NPCs is accompanied by changes in cell appearance, which indirectly reflects changes in the number of cells, namely the decrease of intervertebral disc functional cells and cell activity, mainly due to cell senescence and apoptosis. In this study, although syndecan-4 had effects on the growth and activity of NPCs in the short term, no obvious effects were found in the long term. Even though p53 signaling pathway is closely related to cell growth and apoptosis, it might play a different role under the effect of syndecan-4.

In order to further study the effect of syndecan-4 on the nucleus pulposus of intervertebral disc during degeneration, we detected expression levels of NPC-associated degenerative factors in syndecan-4 overexpressing or knocked-down cells. Western blot and real-time PCR results showed that, in syndecan-4 overexpressing cells, both syndecan-4 protein and mRNA levels increased significantly. However, in syndecan-4 knocked-down cells, protein and mRNA levels were significantly decreased, indicating that our syndecan-4 overexpression and knockdown systems were successfully constructed. Moreover, the protein level of degeneration-associated factors, including collagen II, aggrecan, and Sox-9, decreased when syndecan-4 was overexpressed. Collagen II and aggrecan mRNA levels were also lower. The degeneration indicator collagen X increased in both protein and mRNA levels in the syndecan-4 overexpression group. In contrast, the protein levels of aggrecan and collagen II and the mRNA levels of collagen II, aggrecan, and Sox-9 significantly increased in the syndecan-4 knocked-down cells, while collagen X showed the opposite trend. These results indicate that syndecan-4 may contribute to the degeneration of NPCs.

Saoncella *et al.* showed that, in fibroblasts, syndecan-4 can regulate JNK signaling pathway and the downstream protein levels depending on Rac1, which takes part in the regulation of cell transcription ability 24. It is well known that the JNK signal transduction pathway is an important branch of the MAPK pathway, which plays important roles in a large number of physiological and pathological processes, including cell cycle, proliferation, apoptosis, and cell stress 25. A recent *in vitro* study demonstrated the close relationship between JNK pathway and IVD 26. Thus, we determined whether JNK signaling pathway was affected by syndecan-4 expression in NPCs. Interestingly, syndecan-4 did influence JNK protein in NPCs. Our results show that syndecan-4 overexpression increased JNK protein phosphorylation and total JNK expression levels, while syndecan-4 knockdown clearly reduced JNK protein phosphorylation and total JNK expression levels. This showed that syndecan-4 can activate the JNK signaling pathway in NPCs of intervertebral discs, and that JNK pathway might be involved in the degeneration of NPCs induced by syndecan-4.

The JNK signaling pathway can activate downstream p53 signaling pathways 27-29. Thus, we also examined the protein levels of p53 protein and its target genes in NPCs. Syndecan-4 overexpression significantly increased the expression level of p53 protein and its target genes, MDM2 and p21, in NPCs, indicating that the p53 pathway is activated in these cells. However, whether p53 pathway affects the degeneration process of NPCs remains controversial. Some believe that p53 has a protective effect on NPCs 30, while others have found that p53 is involved in the degradation and apoptosis of NPCs 31, 32. By overexpressing exogenous p53 in NPCs, we found that p53 significantly reduced the protein and mRNA levels of degeneration-associated factors, including aggrecan and collagen II, suggesting that p53 can promote the degeneration of NPCs. In order to verify the effects of JNK signaling pathway and its downstream p53 signaling pathway in the syndecan-4-induced degeneration of NPCs, we treated cells with a JNK inhibitor, SP600125. We found that when the JNK signaling pathway is inhibited, syndecan-4 overexpression can no longer activate the p53 signaling pathway, and that syndecan-4 could no longer inhibit the expression of collagen II and aggrecan. When SP600126 was applied, the protein expression of collagen II and aggrecan is even higher than that in control group. While collagen X showed a even lower result. JNK inhibitor might also inhibit the effect of endogenous syndecan-4 and thus delay the degeneration of NPCs. These results indicate that syndecan-4 affects the degeneration of intervertebral disc NPCs through the JNK/p53 signaling pathway.

With a ChIP assay, we determined that p53 protein can bind to the promoter regions of positive control genes MDM2 and p21 to significantly regulate their transcriptional expression in NPCs. p53 can directly bind and regulate aggrecan transcriptional activity; however, the same is not true for Sox9 or collagen II. Thus, aggrecan is a target gene of p53 protein in NPCs during degeneration. This is consistent with previous studies. Wang *et al.* demonstrated that syndecan-4 promotes the degradation of aggrecan through activating ADAMTS-5, leading to the degeneration of intervertebral discs 16.

We also verified the biological role of syndecan-4 in an *in vivo* experiment. In this experiment, we used a MRI sagittal weighted scan on rabbit postoperative T2 to compare changes in the experimental intradiscal signal before and after operation and to observe the degree of disc degeneration according to the modified Thompson classification method in the evaluation of lumbar degeneration.

At an early stage, the degeneration of intervertebral disc occurs in the nucleus pulposus tissue. The decrease of water and proteoglycan contents is the main pathological change. Signal intensity of MRI T2-weighted images can indirectly reflect the water content of the NP tissue. Eight weeks after injection, MRI results showed that the disc signal strength in the control and saline groups decreased significantly. However, the intervertebral disc high-signal area in the syndecan-4 siRNA group was significantly greater than that of the control and saline groups. Radiology also confirmed the presence of stenosis in degeneration and saline groups, while the syndecan-4 siRNA group presented no obvious stenosis, indicating that no IVD occurred. The Thompson score also showed an obvious lower grading for the syndecan-4 siRNA group. These data suggest that the speed of IVD in the syndecan-4 siRNA group was slow when compared with that in the other two groups. Pathological results supported the MRI results. Hematoxylin-eosin staining showed that, after 8 weeks of treatment in the control and saline groups, the intervertebral disc structure was disordered, and no nucleus pulposus tissue regeneration was observed. The peripheral layered structure organized as a fiber ring in the syndecan-4 siRNA group was normal, the nucleus pulposus tissue was regenerated, and normal NPCs were observed. Immunohistochemistry also showed that the expression of collagen II and aggrecan was significantly increased.

We also confirmed the mechanism underlying the biological role of syndecan-4 *in vivo*. Immunohistochemistry showed that, compared with that in the control group, JNK and p53 expression was significantly decreased in the syndecan-4 siRNA group, while aggrecan expression increased, suggesting that syndecan-4 knockdown could downregulate the residual biological activity of JNK and p53 in intervertebral disc NPCs, effectively promoting NPC matrix component synthesis and secretion.

## Conclusion

Syndecan-4 has not yet been reported as a target for treating early degeneration of the intervertebral disc via injection into the disc itself. In our experiment, using a syndecan-4 siRNA for early intervention in disc degeneration resulted in an increase in NPC numbers and cartilage-like matrix production compared to those in the control group. While collagen X expression decreased, that of aggrecan, collagen II, and Sox-9 increased in the intervention group. The main mechanism underlying the effect of syndecan-4 siRNA treatment was through the inhibition of the JNK/p53 pathway, protecting and promoting the synthesis and regeneration of aggrecan extracellular matrix.

## Figures and Tables

**Table 1:** Primers sequences for qPCR

Primer Name	Primer Sequence
β-actin-F	GGCGGCACCACCATGTACCCT
β-actin-R	AGGGGCCGGACTCGTCATACT
Syndecan-4-F	GGACCTCCTAGAAGGCCGATA
Syndecan-4-R	AGGGCCGATCATGGAGTCTT
Collagen-II-F	GGAAGAGTGGAGACTACTGGATTGAC
Collagen-II-R	TCCATGTTGCAGAAAACCTTCA
Aggrecan-F	CACCTACAAGCACAGGCTACAGAA
Aggrecan-R	AAGGTGCAACGAAGCAGCATGA
Sox-9-F	CGCCATCTTCAAGGCGCTGC
Sox-9-R	CCTGGGATTGCCCCGAGTGC
Collagen-X-F	CCCTTTTTGCTGCTAGTATCC
Collagen-X-R	CTGTTGTCCAGGTTTTCCTGGCAC

## Abbreviations

IVD: intervertebral disc degeneration
NPC: nucleus pulposus cells
ChIP: chromatin immunoprecipitation
MRI: magnetic resonance imaging
